# A Review of Robotic Fish Based on Smart Materials

**DOI:** 10.3390/biomimetics8020227

**Published:** 2023-05-29

**Authors:** Shiwei Ma, Quanliang Zhao, Meixi Ding, Mengying Zhang, Lei Zhao, Can Huang, Jie Zhang, Xu Liang, Junjie Yuan, Xingtao Wang, Guangping He

**Affiliations:** 1 School of Mechanical and Materials Engineering, North China University of Technology, Beijing 100144, China; 17105050124@mail.ncut.edu.cn (S.M.); meixi2333@163.com (M.D.); zhangmengying411@163.com (M.Z.); zhaolei@ncut.edu.cn (L.Z.); zhangjie@ncut.edu.cn (J.Z.); yuanjj@ncut.edu.cn (J.Y.); hegp55@ncut.edu.cn (G.H.); 2 College of Engineering and Technology, Zunyi Normal University, Zunyi 563006, China; zyncwxt@163.com

**Keywords:** biomimetic robot, swimming mechanism, hydrodynamic modelling, smart materials

## Abstract

The present study focuses on summarizing the recent advancements in the field of fish swimming mode research and bionic robotic fish prototypes based on smart materials. It has been widely acknowledged that fish exhibit exceptional swimming efficiency and manoeuvrability compared to conventional underwater vehicles. In the pursuit of developing autonomous underwater vehicles (AUVs), conventional experimental methods often prove to be complex and expensive. Hence, the utilization of computer simulations for hydrodynamic modelling provides a cost-effective and efficient approach for analysing the swimming behaviour of bionic robotic fish. Additionally, computer simulations can provide data that are difficult to obtain through experimental methods. Smart materials, which integrate perception, drive, and control functions, are increasingly being applied to bionic robotic fish research. However, the utilization of smart materials in this field is still an area of ongoing research and several challenges remain unresolved. This study provides an overview of the current state of research on fish swimming modes and the development of hydrodynamic modelling. The application of four distinct types of smart materials in bionic robotic fish is then reviewed, with a focus on analysing the advantages and disadvantages of each material in driving swimming behaviour. In conclusion, the paper highlights the key technical challenges that must be addressed for the practical implementation of bionic robotic fish and provides insights into the potential future directions of this field.

## 1. Introduction

Bionics is a discipline that involves the investigation of the structural and functional characteristics of organisms and the creation of new equipment and tools based on these principles. As land resources become scarce, the ocean, which is characterized by its vast untapped resources and diverse marine life, has drawn increasing attention from researchers and engineers [1]. Given that two-thirds of the Earth’s surface is covered by the ocean, the deep sea is abundant in natural resources and marine life. Furthermore, the ocean’s surface is a rich source of solar and tidal energy. However, the challenging underwater environment, combined with the low energy conversion efficiency, large size, and poor mobility of existing underwater detection and operation devices, presents significant obstacles for underwater operations [2]. To overcome these challenges, researchers have turned to the development of bionic robotic fish, which take inspiration from the efficient propulsion modes of fish that have evolved over millions of years [3,4]. Bionic robotic fish represent a new type of autonomous underwater vehicle (AUV) that integrate hydrodynamics, machinery, electronics, control, and computer technologies [5]. Currently, AUVs are primarily utilized for various applications, including marine observation [6], underwater pipeline inspection [7], environmental protection [8], and rescue operations [9]. However, the design of AUVs for different applications must be tailored to meet specific requirements, such as mobility and endurance for marine observation and concealment for military applications. Despite recent progress, much remains to be discovered in the study of fish swimming mechanisms, and the bionic principles of bionic robotic fish need to be further refined. Additionally, the limited volume and load capacity of AUVs restricts their working time and range, which hinders their widespread use in production and daily life. Thus, there remains significant room for improvement in the development of AUVs.

**Figure 1:**
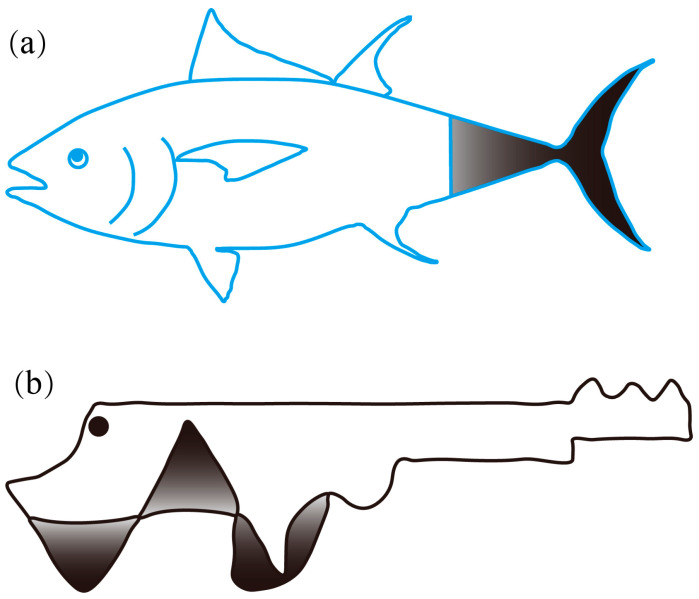
Fish with typical (**a**) BCF and (**b**) MPF swimming modes. (The shaded part is the source of thrust.)

The research on bionic robotic fish can be traced back to 1926 when Breder [10] classified the swimming modes of fish into two categories: body and/or caudal fin swimming (BCF, as shown in Figure 1a) and median and/or paired fin swimming (MPF, as shown in Figure 1b). In 1936, Gray [11] made an estimation that compared to a rigid dolphin model, the muscles of a real dolphin required only one-seventh of the force to maintain the same swimming speed. This observation served as a major inspiration for researchers to investigate the swimming mechanism of fish and sparked interest in the field of bionic robotic fish. In the 1960s, a variety of theories were proposed to explain the swimming mechanism of fish, including the resistive force theory [12] and reactive force theory [13,14,15,16,17]. Among the reactive force theories, three theories have been widely used in hydrodynamic modelling: the elongated body theory (EBT) proposed by Lighthill [13,17] in 1970, the actuator-disc theory proposed by Horlock [16] in 1978, and the wave plate theory (WPT) proposed by Cheng [14,15] in 1994. The development of bionic robotic fish prototypes began with the Robo Tuna and Robo Pike [3,18,19] developed by the Massachusetts Institute of Technology (MIT) in 1995. Since then, significant progress has been made in this field, developing various bionic robotic fish, such as boxfish-like fish [20], robotic manta rays [21], and robotic mackerel [22]. These studies have enhanced our understanding of the swimming mechanism of fish and paved the way for the creation of advanced underwater vehicles.

In order to meet the growing performance demands of AUVs and to propose innovative design methods, a comprehensive review of the swimming modes of robotic fish, hydrodynamic modelling, advanced materials, and actuators is necessary. Previous studies have investigated various aspects of this field, including the hydrodynamics of flapping wings as analysed by Triantafyllou et al. [23]; the control of robotic fish by Colgate et al. [24]; the progress in fluid dynamics, neural-based control, and artificial muscle as summarized by Bandyopadhyay [25]; the kinematics and fluid dynamics of median and paired fins by Kato [26]; the use of smart materials, such as shape memory alloys (SMA), lead zirconate titanate (PZT), and ionic polymer metal composites (IPMCs), by Chu et al. [27]; and the design and analysis of CPG (central pattern generator) model applied to swimming robots by Tsybina et al. [28]. This article aims to provide a comprehensive overview of fish swimming modes and hydrodynamic modelling. Moreover, we introduce some typical advanced materials such as SMA, IPMCs, piezoelectric, and DE, as well as the application of actuators based on these materials in recent AUV prototypes. Finally, the existing challenges in AUV development will be discussed, along with a prospective outlook on the future of this field.

## 2. Hydrodynamic Analysis Model

**Figure 2:**
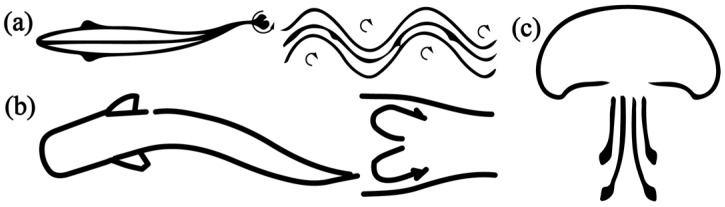
Main propulsion procedures [31]. (**a**) Effect of the tail vortex. (**b**) Effect of inertia. (**c**) Momentum injection.

The proper design of the prototype of AUVs necessitates an in-depth characterization of its hydrodynamic model [24,29,30]. The complexity of the hydrodynamics of fish swimming can significantly impact the accuracy of the modelling, which is a major challenge in hydrodynamic modelling. The forces acting on a fish underwater stem primarily from the friction force and pressure difference created by the pressure gradient. The hydrodynamic motion of fish is primarily influenced by the following factors:

(1) Tail vortex effect (as illustrated in Figure 2a). The vortices generated by the vortical separation in the boundary layer, as well as the backward jet created by the tail vortex, result in the generation of thrust acting on the fish body as the fin swings.

(2) Inertial effect (as illustrated in Figure 2b). The acceleration experienced by the fish when swimming underwater leads to a change in its motion state, which arises from the reaction force created by the change in the water’s momentum. This is referred to as the effect of added mass and is an inertial force. As the fish swings, local inertial forces are generated by all parts of its body, with their combined forces in the forward direction constituting the thrust. This thrust is equal to the product of the mass of the water and the acceleration.

(3) Leading edge suction. As the fish swims and the fluid flows over the leading edge of the caudal fin with a large curvature, the local velocity increases, leading to the formation of a low-pressure area that results in suction at the leading edge, contributing to the thrust.

(4) Momentum injection (as illustrated in Figure 2c). As fluid is ejected from the valve, it injects momentum into the surrounding fluid, generating a force that is proportional to the mass of the ejected fluid and the jet velocity. This force reacts on the fish’s body, constituting a part of the thrust. Generally, jellyfish and other similar organisms mainly rely on momentum injection to generate thrust, but this is not the swimming mechanism of BCF or MPF.

Most fish will be affected by more than two factors at the same time. In order to study the effects of different factors on fish swimming, hydrodynamic modelling and analysis are usually carried out for fish. The current hydrodynamic modelling methods are roughly divided into two categories: the numerical method and the analysis method.

### 2.1. Numerical Method

The numerical method represents a widely used approach for the analysis of fluid flow through the use of computational fluid dynamics (CFD). This method is based on the division of the flow domain into a discretized network of grid elements, which allows for the numerical solution of the governing equations, typically the Navier–Stokes (N–S) equations, within each element. The numerical method provides valuable insights into the flow field by generating a wealth of information, such as pressure and velocity distributions, which cannot be obtained through experimental means.

**Figure 3:**
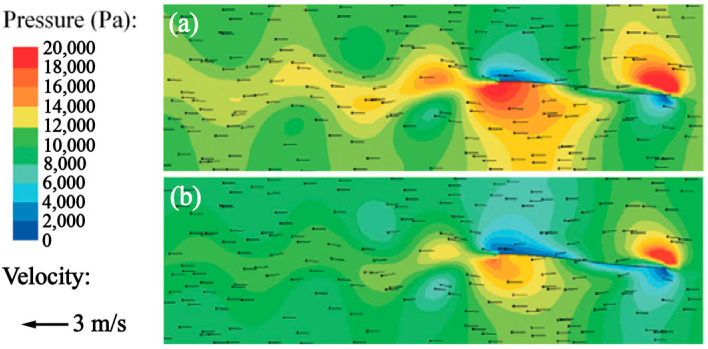
Superimposition of velocity field and pressure field [31]. (**a**) BCF. (**b**) MPF.

In 2012, Lamas et al. [32] conducted a study on the phenomenon of reverse Karman vortex street, which is generated as a result of the periodic shedding of vortical structures behind the caudal fins of swimming fish, as observed in Figure 3a,b. Subsequently, Van Buren et al. [33] verified the impact of this vortex street on both thrust and swimming efficiency and demonstrated that it was possible to control thrust by manipulating the vortical structure of the caudal trace of the pitching plate.

**Figure 4:**
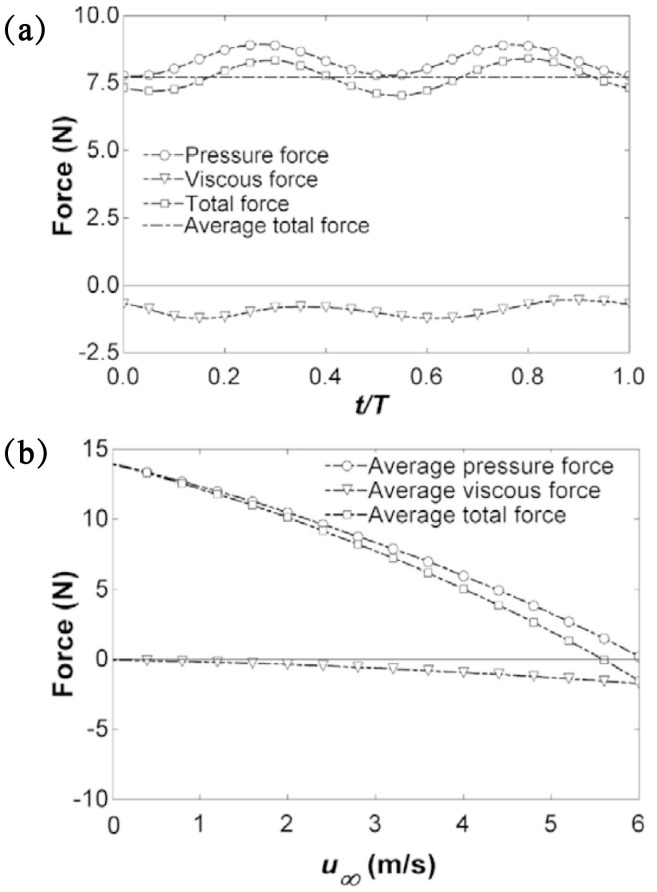
Stress change curve of fish swimming [31]. (**a**) Time variation of the average total, total, instantaneous, and viscous forces. (**b**) Average forces against cruising velocity for anguilliform swimming.

Compared with AUVs, fish exhibit lower energy consumption during swimming due to their ability to mitigate resistance and enhance propulsion efficiency through the suppression of turbulence [34,35,36]. The pressure difference created by the reverse Karman vortex street results in the generation of pressure, given by the following expression

(1) $$F_{p}=-\int p\hat{n}dS$$

where *p* is pressure; *S* is the surface of the fin; $\hat{n}$ is the unit vector perpendicular to the surface. In addition, the viscous (friction) force of water can be expressed as

(2) $$F_{v}=-\int \tau_{ij}\hat{n}dS$$

where *τ_ij_* is the viscous stress tensor. The total force acting on the fish is comprised of pressure and viscous forces, as demonstrated in Figure 4a. The components of these forces in the direction of motion exhibit two peaks per cycle, reflecting the forward and backward strokes of the caudal fin. The presence of a positive total force results in the acceleration of the object, with the added mass effect being further enhanced by the system’s inertia. The relationship between average force and speed depicted in Figure 4b shows that when the speed is zero, the pressure force is at its maximum and the viscous force is at its minimum. As the velocity increases, the pressure decreases and the viscous force increases. Once the speed reaches a point where the pressure and viscous force are equal, the total force becomes zero and the speed remains constant.

In bionics, non−dimensional numbers are often used to describe the involved parameters [37]. For laminar fluid and Newtonian fluid, their flow motion is given by the N–S equation of mass and momentum conservation

(3) ∇⋅u=0

(4) $$\frac{\partial u}{\partial t}+\nabla \cdot (\mathrm{uu})=-\frac{\nabla p}{\rho }+v\nabla^{2}u+g$$

where ***u*** is the velocity; *p* is the pressure; *ρ* is the density; *v* is the kinematic viscosity; ***g*** is the gravity. The above parameters are converted into non-dimensional numbers through the reference parameters *L_ref_*, *u_ref_*, *p_ref_*, *t_ref_*, and *g_ref_* of length, velocity, pressure, time, and gravity, respectively, as shown in Table 1.

Equations (3) and (4) can be converted into

(5) $$\frac{1}{L_{ref}}\nabla^{*}\cdot (u^{*}u_{ref})=0$$

(6) $$\begin{array}{l} \frac{1}{t_{ref}}\frac{\partial (u^{*}u_{ref})}{\partial t^{*}}+\frac{1}{L_{ref}}\nabla (u^{*}u_{ref}u^{*}u_{ref})\\ =-\frac{1}{\rho }\frac{1}{L_{ref}}\nabla^{*}(p^{*}p_{ref})+v\frac{1}{L_{ref}^{2}}\nabla^{*2}(u^{*}u_{ref})+g^{*}g_{ref} \end{array}$$

In Table 1, *L* is the length of the fin, *U* is the cruise speed, and the mass conservation equation and momentum conservation equation are as follows

(7) $$\frac{1}{L}\nabla^{*}\cdot (u^{*}U)=0$$

(8) $$\frac{U}{L}\frac{\partial (u^{*}U)}{\partial t^{*}}+\frac{1}{L}\nabla \cdot (u^{*}Uu^{*}U)=-\frac{1}{\rho }\frac{1}{L}\nabla^{*}(p^{*}\rho U^{2})+v\frac{1}{L^{2}}\nabla^{*2}(u^{*}U)+g^{*}$$

The Reynolds number is expressed as *Re = UL/v*, and the Froude number is expressed as *Fr = U/*$\sqrt{Lg}$ to obtain the non-dimensional control equation

(9) $$\nabla^{*}\cdot u^{*}=0$$

(10) $$\frac{\partial u^{*}}{\partial t^{*}}+\nabla^{*}\cdot (u^{*}u^{*})=-\nabla^{*}p^{*}+\frac{1}{Re}\nabla^{*2}u^{*}+\frac{1}{Fr^{2}}g^{*}$$

The Reynolds number and Froude number are crucial determinants in the hydrodynamics of AUVs. The frequency of the movements is commonly expressed as a non-dimensional Strouhal number, *St = fA/U*, where f represents frequency and *A* represents amplitude. Research has indicated that the Strouhal number plays a role in the formation of vortices, with typical values ranging from 0.2 to 0.4. Studies also suggest that optimal vortex generation occurs within this range of Strouhal numbers.

The application of the numerical method has proven to be a crucial tool for the advancement of fluid mechanics, providing a comprehensive understanding of fluid flow behaviour in a wide range of real-world scenarios. Furthermore, the continual improvement of numerical algorithms and computational resources has led to a substantial increase in the accuracy and reliability of CFD simulations, making this method a highly desirable tool for engineers and scientists alike [38,39,40,41,42,43,44].

### 2.2. Analysis Method

It is a common practice to employ numerical methods for solving the N–S equation in order to study the hydrodynamic interaction of fish flexible structures. The linear Euler–Bernoulli beam model can provide an accurate simulation of this interaction, but the computational demands are substantial. In contrast, the analytical modelling method is more practical.

In the field of hydrodynamic force on a swimming fish, two distinct theories have been proposed to explain this phenomenon. The first, referred to as the resistive force theory, was introduced by Taylor [12]. This theory utilizes the steady flow theory to calculate the hydrodynamic force acting on a fish at a given point in time. It breaks down the object into numerous infinitesimal parts, each of which generates both thrust and resistive force. When the fish moves, the resistive force perpendicular to its body’s direction is found to be greater than the resistive force parallel to its forward direction, thus resulting in a net thrust in the forward direction. However, this theory has limitations, as it neglects the effect of inertial forces and oversimplifies the shape of the fish body, making it only applicable to cases of low Reynolds numbers. The second theory, referred to as the two-dimensional wave propulsion theory, was proposed by Wu [14]. This theory considers the fish body as an elastic thin plate with infinite height and waves and draws analogies to the study of vibrating wings in air to analyse the motion of the fish. This method, along with the elongated body theory in aerodynamics, has become the foundation for the elongated body theory proposed by Lighthill.

Lighthill [13,45] initially proposed the EBT for deformable bodies in the early stages of his research. The EBT accounted for the effect of added mass and approximated the impact of wake dynamics through the kinetic momenta balance in a control volume surrounding the fish body. The theory laid out the conditions for an elongated fish to achieve high propulsion efficiencies, such as a forward speed that is 20% lower than the body’s swing speed, an increasing amplitude of the swing from the fish’s head to the maximum value at the caudal fin, an elongation of the caudal fin exceeding a certain critical value, and the inclusion of positive and negative values in the swing phase to balance the lateral force of water. Lighthill [17,45] later introduced the large amplitude elongated body theory (LAEBT), which extended the scope of the EBT to cases of large amplitude deformation. The LAEBT was applied to analyse anguilliform and carangiform swimming styles. According to this theory, the fluid around the fish is first the steady flow around the object, and then the flow generated by the displacement *h(x,t)*. The relationship between the flow component *V(x,t)* on a given cross-sectional area *S_x_* and the fluid velocity *U* is as follows

(11) $$V(x,t)=(\frac{\partial h}{\partial t})+U(\frac{\partial h}{\partial x})$$

The theory uses partial differential equations to calculate the thrust and efficiency of fish body swing when swimming with an irregular symmetrical rhythm. The expression of *h(x,t)* is introduced

(12) $$h(x,t)=f(x)\times g(t-\frac{x}{c})$$

where *f*(*x*) is the swing amplitude, *g*(*x,t*) is the wave function of the oscillation frequency, and *c* is the swimming speed of the fish [46,47]. Lighthill first introduced Newtonian fluid medium into the EBT. Later, a travelling wave model was proposed to describe BCF motion

(13) $$y(x,t)=[c_{1}x+c_{2}x^{2}][sin(kx+\omega t)]$$

where *y* and *x* are lateral displacement and forward displacement respectively; t is time; *c_1_* and *c_2_* are linear and quadratic wave amplitudes; *k* is volume wave number; *ω* is wave frequency.

These four types of propulsion forces that generate forward motion include the *y*-axis lateral force and the *x*-axis thrust. The lateral force also generates energy loss while helping fish to yaw and sideslip. In Lighthill’s EBT, it is necessary to subtract the kinetic energy rate of lateral motion loss to calculate the average thrust of fish

(14) $$\bar{T}=[\frac{1}{2}\rho A(l)(\bar{{\left(\frac{\partial y(x,t)}{\partial t}\right)}^{2}}-U^{2}\bar{{\left(\frac{\partial y(x,t)}{\partial x}\right)}^{2}})]$$

Among them, the most typical is anguilliform. Because its fluctuation amplitude increases from beginning to end, its hydrodynamic force is low when swimming with high manoeuvrability, resulting in more energy loss. Taylor [12,48,49] discussed the influence of some aspects of swimming propulsion. In the first two papers, the problem of the influence of microorganism swimming in viscous effect played a leading role [48,49]. In the third paper, he studied the swimming theory of long and narrow animals [12].

(15) $$y=Bsin\frac{2\pi }{\lambda }\{x+(U-V)t\}$$

Compared with the first two theories, Lighthill’s theory has higher computational efficiency, easily estimated hydrodynamic parameters, and multiple degrees of freedom Lighthill wave functions generate different swimming modes. Generally, it is the standard motion model for AUVs, providing a theoretical model basis for the development, analysis and modelling of AUVs.

In short, compared with the other three theories, the resistance theory believes that viscosity plays a leading role in motion, and ignores the impact of inertial force, so it is only applicable to long narrow fish with low Reynolds number. However, wave plate theory is applicable to the fish in the form of a two-dimensional flat plate, and the fluid is simplified as an inviscid incompressible fluid. The elongated body theory is applicable to slender fish, that is, each individual bony ray makes only a moderately small angle with the backbone. It proposes the conditions for the slender fish to achieve high-speed propulsion. Furthermore, large amplitude elongated body theory is applicable to fish with large amplitude tail fin swing. It considers the flexure and lateral velocity of fish and estimates recoil.

**Figure 5:**
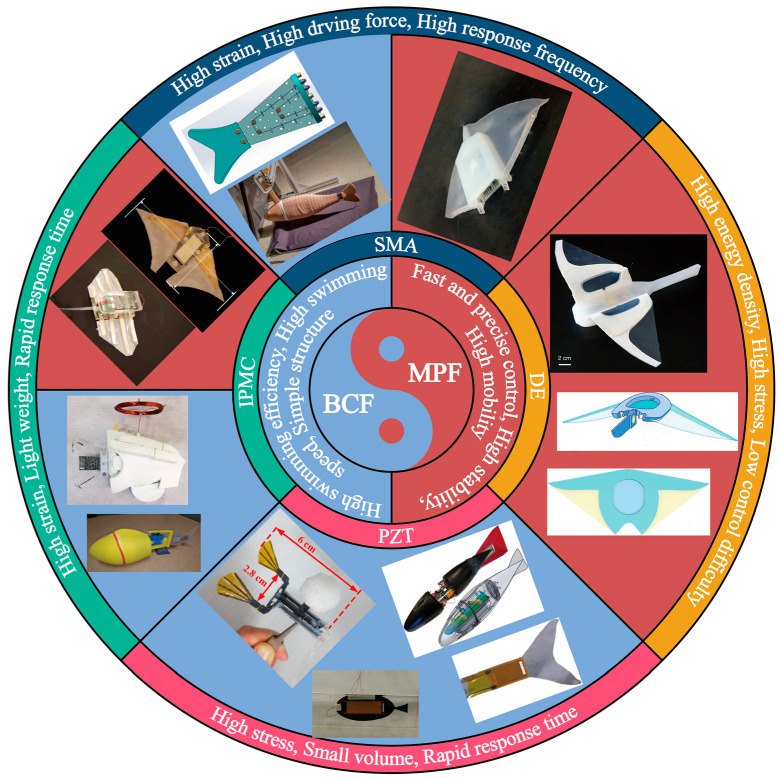
Classification of robotic fish actuated by smart materials. (The relevant images in the figure have been cited in the following text.)

As shown in Figure 5, the swimming modes of fish can be divided into two types: BCF and MPF. Among them, the BCF mode has high swimming efficiency and fast swimming speed, while the MPF mode has more advantages in manoeuvrability and control accuracy. In the following text, the bionic robotic fish is classified according to the different smart materials used by the actuator. At present, smart materials widely used in bionic robotic fish mainly include shape memory alloys, ionic polymer metal composites, piezoelectrics, and dielectric elastomers. They each have different advantages and disadvantages, which will be analysed together with the introduction of the prototype in the following text.

## 3. BCF

The BCF mode can be further classified into four subtypes: anguilliform, subcarangiform, carangiform, and thunniform [50]. The anguilliform mode is characterized by constant amplitude oscillations along the entire body. This mode is highly flexible and offers exceptional manoeuvrability and acceleration capabilities, as well as the ability to swim backwards. However, its swimming efficiency is lower compared to other modes. The subcarangiform mode is similar to the carangiform mode, with a key distinction being a greater level of trunk involvement in the motion for subcarangiform swimming. These two modes are more efficient than anguilliform, and are, therefore, adopted by a larger number of fish. The thunniform mode, which is the most widely used type in nature, is characterized by high swimming efficiency. Approximately 90% of the thrust is generated by the caudal fin, with the remainder generated through the added mass process [51].

### 3.1. SMA

**Figure 6:**
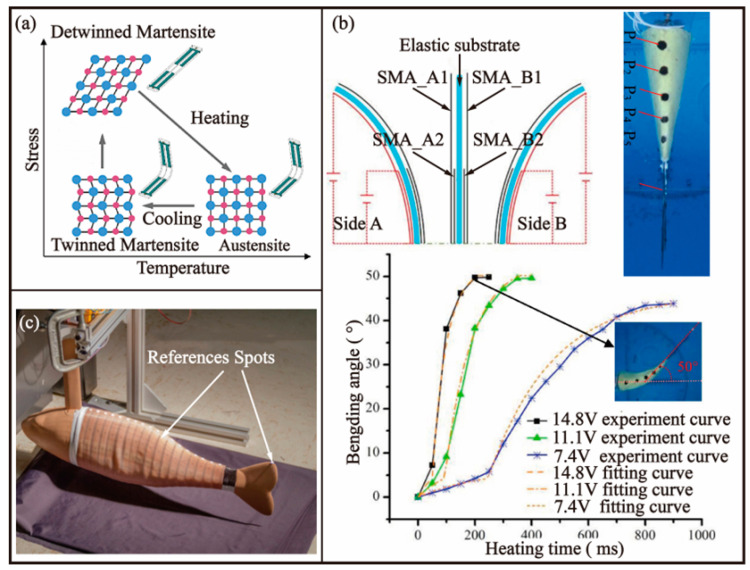
Bionic robotic fish-based BCF mode driven by SMA. (**a**) The principle of SMA [53]. (**b**) Soft fishtail inspired by the Carassius auratus [54]. (**c**) Gish-like robot inspired by the black bass [56].

The phase transition of shape memory alloy (SMA) material is influenced by both temperature and internal stress, exhibiting thermodynamic characteristics and the shape memory effect (SME) under specific conditions [52,53]. As depicted in Figure 6a, the austenite phase transforms into the twinned martensite phase upon cooling and then into the detwinned martensite phase upon application of stress, constituting a positive transformation. Upon heating the martensite phase, the forward transformation deformation is reversed, leading the material to return to its original shape, a process referred to as inverse transformation. This transformation process enables SMA to generate high levels of stress (up to 200 MPa) with low voltage (approximately 2 V) application [54], making it a suitable actuator. The aquatic environment of AUVs is particularly advantageous for cooling SMA and achieving higher frequency.

In a study published in 2019, Li et al. [55] utilized the SMA to drive a bionic flexible fishtail structure that imitates the muscle and bone structure of crucian carp, combined with the natural fish body shape, under the BCF mode. This design allows for flexible movement with a large amplitude. The construction details are depicted in Figure 6b. The heated layer is comprised of a 0.3 mm thick polyvinyl chloride (PVC) material, with EON-01 silica gel serving as the skin and filler for flexibility and insulation to imitate real muscle. The fishtail utilizes the thermodynamic properties of the material to adjust its fluctuations by varying the heating time. The final propulsion frequency of the bionic fish tail was recorded as 0.8 Hz, with a heating time of 80 ms, a bending angle of 22°, and an average propulsion force generated of 0.041 N. Many AUVs that emulate the BCF mode in nature typically utilize multi-joint or flaky tails to achieve flexible motion. However, incorporating such tails into the head region proves challenging, hindering the formation of a streamlined overall design.

In 2015, William et al. [56] developed an SMA-driven deformable fish tail for a robotic fish that emulates the motion of a largemouth bass. The design incorporated a bending structure composed of four pairs of SMA wires, which, when activated by a constant voltage, resulted in symmetrical bending of the tail fin with a maximum bending angle of 170°. The robotic fish was equipped with a silica gel attached to the fins to mimic the skin of a real fish. However, over time, the silica gel may peel off from the edge, limiting the endurance and swimming speed of the robotic fish.

In 2018, William et al. [57], from the Mechatronics Laboratory of the University of Madrid, proposed a novel design for a SMA linear drive robotic fish, inspired by the anatomy and behaviour of black bass (as shown in Figure 6c). By integrating bionic kinematics with electromechanical drive technology, the team created a robotic fish with a flexible trunk and deformable structure, as well as a bionic skin made of liquid silica gel and fibre mesh that used flexible sensors to perceive its environment. The maximum bending degree of the SMA conductor was approximately 38°, and the application of currents higher than 410 mA reduced the shrinkage time, enabling the robotic fish to exhibit a doubled pulling force and fast response. In comparison to SMA fishtail-driven AUVs, this design demonstrated superior swimming efficiency and performance. However, it was noted that the actuator experienced fatigue over time and had reduced strain capacity, highlighting the need for further improvements to reduce current disturbance and vorticity in order to enhance the endurance of these types of AUVs.

In 2021, M. Muralidharan et al. [58] developed a subcarangiform AUV utilizing SMA technology. The skeleton of the bionic robotic fish was fabricated utilizing an acrylonitrile-butadiene-styrene (ABS) polymer, using a 3D printing process. The driving element of the SMA technology was realized by a symmetrical arrangement of a NiTi alloy spring located at the joint of the O-ring. The weight of the AUV was found to be 416 g, with a maximum length of 25 cm. The maximum swing angle in aqueous environments was recorded at 50°, with a maximum stress of 0.39 N. The forward velocity of the AUV was observed to be 24.5 mm/s, generated by the sequential deformation of the SMA technology. However, the design limitations of the AUV presented the issue of lacking the capacity to carry its own battery and control circuit, requiring an external power supply for autonomous operation.

### 3.2. IPMCs

**Figure 7:**
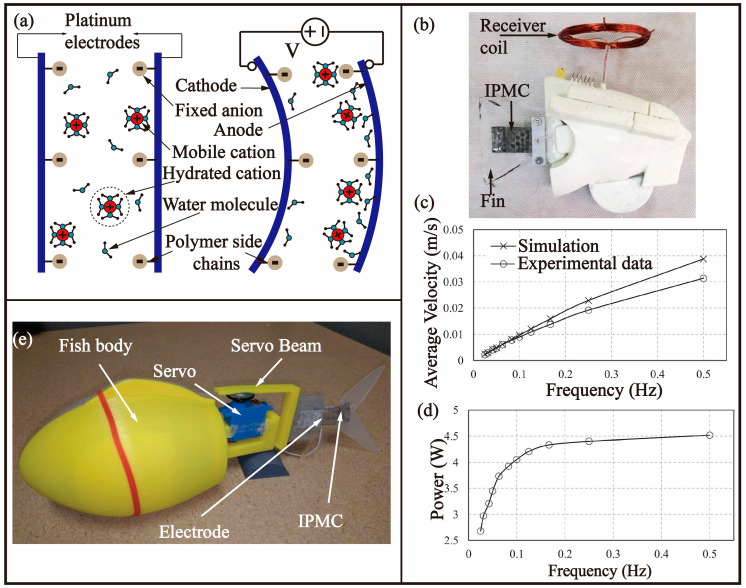
Bionic robotic fish-based BCF mode driven by an IPMC. (**a**) The principle of IPMCs [59]. (**b**) Bionic robotic fish inspired by Scorpis Georgiana fish [60]. (**c**) The average velocity of the robotic fish versus frequency applied to the IPMC [60]. (**d**) Measured power consumption of the robotic fish versus the frequency of the applied voltage to the IPMC [60]. (**e**) Robotic fish propelled by two-joint hybrid fish tail [61].

In the field of polymer-based electro-stimulated actuation, IPMC has emerged as a promising material due to its unique electro-stimulated bending behaviour. As shown in Figure 7a, IPMCs are constructed from a Nafion or Flemionpolymer matrix film that serves as a matrix and conductive metal electrodes that impart electrical conductivity. Upon the application of external electric stimulation to the electrodes, the IPMC material experiences an electric field within its polymer matrix, causing the migration of sodium ions and water molecules towards the cathode. This movement is a result of the unequal distribution of water molecules due to the fixed SO_3-_ ions, leading to a bending effect in the material. The chemical structure of the IPMC changes and mechanical deformation occurs, allowing for its utilization in various applications that require flexible yet robust actuation [59].

In 2022, Hubbard et al. [60] presented a photovoltaic-powered IPMC-driven robotic fish, designed to monitor pollutants in aquatic ecosystems as depicted in Figure 7b. The robotic fish, with a width of 50 mm and a weight of 100 g, was equipped with a wireless energy transmission system that utilized a solar panel for power generation. The solar panel, mounted on the robotic fish, was capable of providing a stable voltage of 12 V and a power output of 10 W (Figure 7d), with an energy conversion efficiency of 5%. The maximum swimming speed of the robotic fish was measured to be 30 mm/s (Figure 7c), which was faster than other that of the single IPMC tail fin-driven robotic fish. However, the inclusion of an efficient solar cell increased the cost of the robotic fish, limiting its speed and mobility, as it was unable to provide energy during night-time conditions.

In 2019, Chen et al. [61] developed a robotic fish propelled by a two-joint hybrid tail, as shown in Figure 7c. The fish shell was printed with ABS material using a 3D printer. The first joint was driven by a DC servo motor, which could produce swift flapping motion; The second joint was driven by IPMC, which could control the forward direction by changing the shape of caudal fin. This structure combined the advantages of high torque of servo motor and the flexibility of IPMC to realize fast forward swimming and small radius steering. In terms of control, the bionic robot fish was equipped with a microcontroller with a Wi-Fi module, and a state-space model was developed to capture the interaction between the tail and the fluid, the driving dynamics of IPMC and the motion dynamics of rigid bodies. The total weight of the built-in battery and control circuit was 20 g, and the driving current of 2A was provided to IPMC through the H-bridge. Finally, the length of the fish including the tail was around 27 cm, and the weight was about 180 g. It could reach a forward speed of up to 45 BL/s (12 cm/s) and turning speed up to 40 deg/s. In the same year, a collision cone approach was adopted to solve the problem of autonomous cruise of bionic robot fish [62]. Using a Lyapunov-based approach, analytical expressions of nonlinear energy- optimal guidance laws for cooperative collision avoidance are determined. Conditions under which it is guaranteed that these laws will achieve successful collision avoidance even in the presence of imperfections in sensor measurements are established.

However, when a constant voltage was applied to the IPMC, it exhibited a phenomenon known as “back-relaxation,” which manifested as an increase in the bending angle of the IPMC over a brief period of time, followed by a gradual decrease after reaching a peak. This effect had a significant impact on the steering dynamics of the robotic fish. In 2021 [63], the phenomenon of back-relaxation in IPMCs was investigated and the previous collision model was refined. The back-relaxation occurs due to the ingress of water molecules from the external environment, which results in the neutralization of the unbalanced water concentration in IPMCs and a consequent bending of the material towards the anode side. This behaviour is largely influenced by the driving voltage of IPMCs and the salinity level of the water. Through experimentation, it was found that a lower driving voltage reduced the magnitude of back-relaxation, but resulted in a slower response time and a reduced bending moment. Meanwhile, the higher the salinity level, the smaller the bending angle generated by IPMCs, which means that the displacement and driving force generated are smaller. To address these limitations, a data-driven approach was employed to model the effect of back-relaxation, resulting in an empirical transfer function. This transfer function was subsequently integrated into the existing collision avoidance control law to formulate a new, improved collision avoidance algorithm. The efficacy of the proposed approach was validated through experiments with two bionic robotic fish.

### 3.3. Piezoelectric

The utilization of the inverse piezoelectric effect in piezoelectric materials is primarily leveraged to drive AUVs. The application of an electric field in the direction of polarization results in the deformation of the material. The removal of the electric field results in the disappearance of the deformation. The inverse piezoelectric effect is demonstrated in Figure 7a. Upon application of a high-strength voltage to the piezoelectric material, it was observed that the deformation is smaller compared to SMA (at around 0.2%); however, the driving stress is significantly higher (approx. 110 MPa), leading to improved accuracy. Furthermore, the material can be made to exhibit bidirectional expansion and contraction and operate at a higher swing frequency.

**Figure 8:**
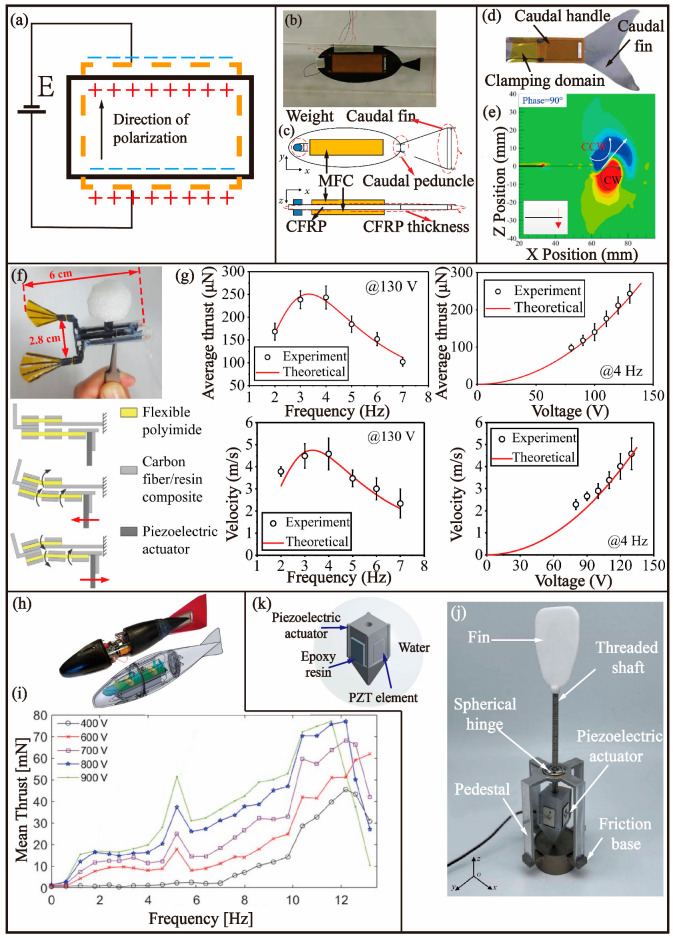
Bionic robotic fish-based BCF mode driven by PZT. (**a**) The principle of PZT. (**b**) Robotic fish driven by MFC [64]. (**c**) Structure of the soft robotic fish [64]. (**d**) Bionic caudal fin inspired by koi fish [65]. (**e**) Computed instantaneous vorticity contours around the propulsion tip at the centre plane [65]. (**f**) Micro-robotic fish with double caudal fins and the movements of four-bar linkage transmission [66]. (**g**) Simulation and experiment of the propulsion force and propulsion speed of micro-robotic fish [66]. (**h**) Trout-like robotic fish [68]. (**i**) Mean thrust frequency response for different actuation voltage amplitude levels over a frequency range [68]. (**j**) A 3-DoF piezoelectric robotic pectoral fin [69]. (**k**) Enclosure modal of the piezoelectric actuator [69].

In 2018, Zhao et al. [64] conducted an analysis of a subcarangiform fish using the acoustic fluid-structure method and developed a bionic robotic fish driven by a piezoelectric ceramic fibre embedded in an epoxy resin (Figure 8b). The soft actuator of the robotic fish was comprised of two polyimide films with interdigital electrodes and a rectangular plate sandwiched in the middle. The body of the robotic fish was made of a combination of a carbon fibre-reinforced polymer (CFRP) plate, two soft actuators, and a steel counterweight in the head, which served to increase the tail displacement, as depicted in Figure 7c. The researchers optimized the structure of the robotic fish through coupling analysis and numerical simulation, resulting in a maximum tail-end displacement of 62 mm. The driving force behind the movement of the robotic fish was primarily generated through the bending deformation caused by the resonance of the combination of the macro-fibre composite (MFC) and CFRP. Turning was achieved by applying asymmetric signals to the soft actuators on either side. When the voltage frequency applied was 15 Hz, the robotic fish was able to generate a maximum speed of 0.6 m/s, a right-turning speed of 51.6 deg/s, and a left-turning speed of 53 deg/s.

In 2019, Luo et al. [65] of Ningbo University conducted a study to develop a bionic miniature fishtail driven by an MFC actuator (Figure 8d). The inspiration for this study was drawn from the koi carp, and its tail fin served as the basis for the creation of a simplified model. Through the use of computational fluid dynamics, the three-dimensional flow generated by the tail fin oscillating propulsion system was analysed. A tail fin prototype was subsequently manufactured using the results of the analysis. The tail fin consisted of an 1106 aluminium alloy base layer, MFC laminates on both sides, and an epoxy resin coating applied to the MFC surface to enhance its bending and waterproofing capabilities. The MFC laminate was composed of a piezoelectric ceramic fibre, an epoxy resin matrix, and interdigital copper electrodes. The maximum underwater oscillation velocity of the bionic fishtail was 248.2 mm/s, with a maximum instantaneous thrust of 21.5 mN and a maximum average thrust of 9.5 mN. As depicted in Figure 8e, based on CFD simulation analysis, the maximum instantaneous thrust is achieved when the phase angle is at 90°, which corresponds to no deflection and negative z-direction velocity. Moreover, the vortex generated by the bionic pectoral fin exhibits markedly uneven spatial distribution on different horizontal planes at this specific phase angle.

In 2021, Liu et al. [66,67] of North China University of Technology developed a lightweight piezoelectric double-tailed micro-robotic fish, as depicted in Figure 8f. The authors utilized advanced manufacturing techniques to construct the fish, which was driven by piezoelectric actuation. As shown in Figure 8g, The experiments conducted revealed that the fish was capable of achieving a maximum thrust of 243 μN when driven by a voltage of 130 V at 4 Hz. Additionally, the maximum average speed of the fish was found to be 4.5 cm/s, while also exhibiting the ability to turn while swimming at high speeds. The total efficiency of the system was calculated to be 1.69%, which represents a significant improvement over other similar AUVs.

In 2021, David et al. [68] of the Georgia Institute of Technology conducted a simulation of AUVs modelled after rainbow trout and powered by MFC actuators, as depicted in Figure 8h. The MFC actuator employed in the study consisted of a flexible fibre piezoelectric structure, composed of PZT fibre and epoxy resin, with interdigital electrodes embedded in Kapton film. This design offers a balance between deformation and driving force, with a maximum driving force of over 10 mN. The mean thrust frequency response is shown for different actuation voltage levels in Figure 8i. The use of MFC as a new piezoelectric material eliminates the need for a complex gear mechanism to transmit propulsion power, enabling the miniaturization of the micro-robot, while also ensuring high performance. The simulation results showed that the rainbow trout-like robotic fish could swim at a maximum speed of 0.85 BL/s in still water when driven at a 5.6 Hz frequency. In a water flow of 1 m/s, the swimming speed was found to be 0.71 BL/s when powered by an external supply and 0.92 BL/s when powered by a battery.

In 2022, Liu et al. [69] presented a novel approach to the design and development of a three-degree-of-freedom piezoelectric pectoral fin for robotic fish, as depicted in Figure 8j,k. This design was based on a combination of CFD simulation and experimental investigation. Through computational fluid dynamics, the researchers analysed the mechanical properties of the pectoral fin and verified the simulation results through experimental means. The experimental results demonstrated the capability of the pectoral fin to produce a maximum thrust of 24.7 N and a response time of more than 10 ms. Furthermore, the authors proposed a control strategy for the pectoral fin of the robotic fish to achieve accurate control of its movement. The proposed design, as well as its control system, exhibited high performance and efficiency, pointing towards a promising future for the development of bionic robotic fish.

### 3.4. DE

**Figure 9:**
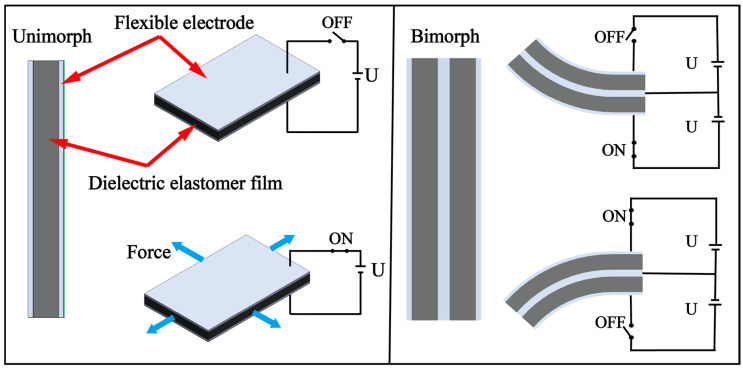
The principle of DE.

In the field of micro-robotics, dielectric elastomer actuators (DEAs) have been identified as promising candidates for soft actuation due to their high elastic modulus (about 1 MPa) and high energy density. As shown in Figure 9, the DEA consists of a middle layer made of a dielectric elastomer film and flexible electrodes positioned above and below the film. When a kilovolt voltage is applied to the flexible electrodes, molecular dipoles are rearranged in response to the electric field, resulting in the accumulation of positive and negative charges on the upper and lower surface electrodes of the film. The resulting Maxwell stress causes compression and thinning of the film, but the hyperelastic material remains uncompressed and results in expansion deformation. The DEA’s high efficiency (theoretical maximum electromechanical efficiency is 90%) and fast response time (response time < 200 μs) make it an ideal candidate for use as a soft actuator in micro-robotic fish.

In 2018, Berlinger et al. [70] from Harvard University developed a stacked driving fishtail using DE materials to allow for autonomous underwater navigation by robotic fish. The unimorph is composed of three active dielectric layers, four electrodes and two encapsulating layers. The tail fin is composed of two unimorphs and flexible adhesives to form a full bimorph. The swing motion of the tail fin can be realized by alternately switching the unimorph on both sides of the actuator. The robotic fish was equipped with a lithium-ion battery, a pressure sensor, an SD card reader, and a circuit structure that provides a high drive voltage for the DE. The robotic fish measured 100 mm in length and 115 g in weight, and demonstrated the capability to swim in a horizontal plane at a speed of 0.55 mm/s and in a vertical dive at a speed of 30 mm/s. However, the researchers noted that when the drive voltage was too high, the DE layer was prone to superposition, increasing the thrust but also elevating the risk of electrolyte breakdown.

## 4. MPF

The swimming speed of MPF in fish generally falls within the range of 3 BL/s and relies on the generation of thrust through fin fluctuation. MPF can be classified into wave-type and oscillation-type, further subdivided into rajiform, diodontiform, amiiform, gymnotiform, balistiform, labriform, and tetraodontiform [52]. Fish that swim in a rajiform manner possess intricate fin structures, and the fluctuation amplitude at the top of their front fins is relatively high, with a gradual reduction towards the front as they flap laterally. Manta rays and rays both swim in this mode. The diodontiform displays at least two complete wavelengths of pectoral fins in its swimming pattern. The amiiform and gymnotiform use undulating dorsal and anal fins, respectively, to generate thrust. The tetraodontiform, as found in sunfish, results from the cooperative undulation of the dorsal and anal fins. The balistiform, seen in trigon fish, features a flat body and an inclined middle fin, which generates thrust through the combined fluctuation of the anal and dorsal fins. The oscillation of MPF involves the fluctuation of the median and/or paired fins, making it a more challenging mode to imitate.

### 4.1. SMA

**Figure 10:**
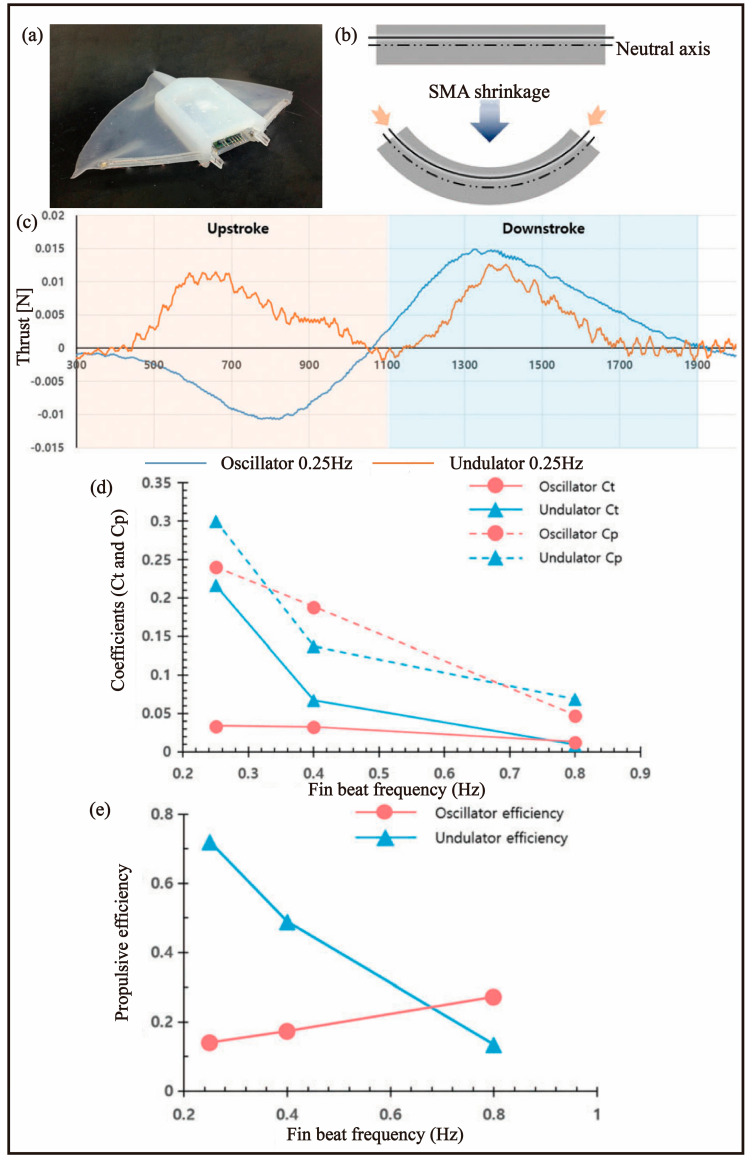
Ray−like robotic fish [71]. (**a**) Prototype. (**b**) Deformation principle. (**c**) Thrust tendency in one cycle for fin beat frequencies of 0.25. (**d**) Thrust coefficients (thrust coefficient: Ct; power coefficient: Cp). (**e**) Propulsive efficiency.

In 2016, Kim et al. [71] developed a micro-propulsive fish-based MPF AUV that utilized a pectoral fin-based propulsion mechanism, modelled after the anatomy of rays, as depicted in Figure 10a. The pectoral fin of rays consists of multiple radial cartilage structures and muscles attached along the body’s radial axis. The team leveraged the structural and functional characteristics of ray pectoral fins as a reference and employed SMA and an embedded directional support to deform the pectoral fin and generate thrust by shortening the SMA line (Figure 10b). The resulting robotic fish demonstrated a thrust of 15 mN (Figure 10c) and a swimming speed of 0.26 BL/s with the pectoral fin beat frequency of 0.25 Hz, resulting in a 38% improvement in efficiency compared to existing designs. According to Figure 10d, the undulator and oscillator will have obvious differences when they oscillate at low frequencies. The oscillator generates opposite thrust on the upstroke, and according to Figure 10e, it can be considered that the passive fluctuation of the oscillating fin can generate thrust more effectively. However, the lack of a buoyancy control module hindered the autonomous swimming capabilities of the robotic fish.

### 4.2. IPMCs

**Figure 11:**
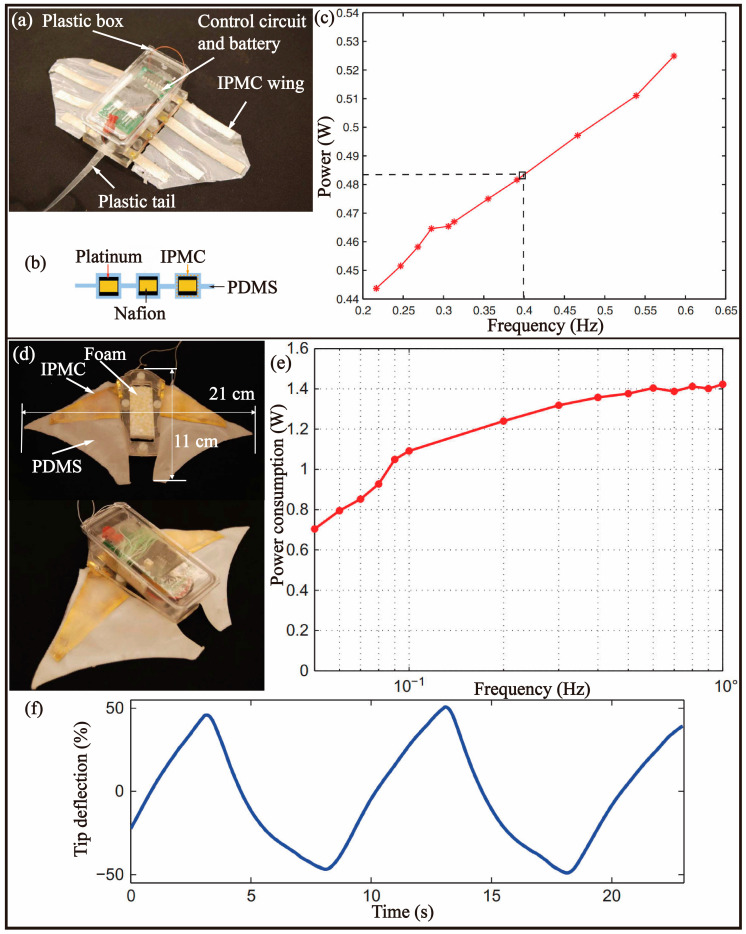
Bionic fish-based MPF mode driven by an IPMC. (**a**) Robotic fish with 3D kinematic pectoral fin [59]. (**b**) Structural composition of pectoral fin [59]. (**c**) Power consumption versus frequency [59]. (**d**) Robotic fish inspired by manta rays [72]. (**e**) Power consumption versus operating frequency [72]. (**f**) Tip deflection of pectoral fin [72].

In 2011, Chen et al. [59] utilized a synthetic approach to integrate the IPMC beam and PDMS to fabricate an actuator. By individually controlling the IPMC beam, the actuator was capable of generating complex three-dimensional movements, such as oscillation and fluctuation, and was installed on a manta ray-inspired bionic robotic fish, as illustrated in Figure 11a. The actuator was comprised of four IPMC beams and a 190 μm thick PDMS layer, which was improved in terms of driving performance through a one-step multiple platinum plating process aimed at reducing the electrode surface resistance (Figure 11b). The bionic robotic fish consisted of two symmetrically arranged actuators, along with a control unit equipped with a PCB board and lithium battery, mounted on the fish. According to experiments, the bionic robotic fish was able to swim at a speed of 0.42 cm/s (0.053 BL/s) with a power consumption of 1 W (Figure 11c). However, the voltage limit of 6 V imposed by the IPMC material resulted in a limited swimming speed, and the absence of a control strategy limited its functionality to simple swimming.

In 2014, Chen et al. [72] investigated the feasibility of utilizing an improved IPMC actuator for the development of bionic robotic fish. The earlier approach of installing four IPMC beams on a polydimethylsiloxane (PDMS) film to achieve 3D structural changes posed significant challenges in terms of control, prompting the researchers to reduce the number of IPMCs to a single unit for ease of control, as depicted in Figure 11d. A layer of gold coating was also added to enhance conductivity. The resulting artificial pectoral fin demonstrated improved performance, with a 100% tip deflection (Figure 11f) and 40° torsion angle. During the testing of a bionic robotic fish prototype, the researchers observed a swimming speed of 0.74 cm/s (0.067 BL/s) and a power output of 2.5 W (Figure 11e). However, the prototype model had not undergone kinematic and dynamic simulations or shape structure optimization, leaving room for further improvement in its propulsion efficiency and speed.

In 2014, Hubbard et al. [73] presented a novel design for a bionic robotic fish that employed a combination of tail-swinging propulsion and fin control, as depicted in Figure 11g. The propulsion mechanism was achieved by using two distributed IPMC actuators in each pair of fins. The application of different voltage levels resulted in torsional deformation of the fin surface, thereby enabling control of the direction of motion. The robotic fish was capable of reaching a swimming speed of 28 mm/s (Figure 11h). However, the authors noted that at low driving frequencies, the driving amplitude of the tail was substantial, leading to a larger yaw angle of the robotic fish and limiting its forward speed.

### 4.3. DE

**Figure 12:**
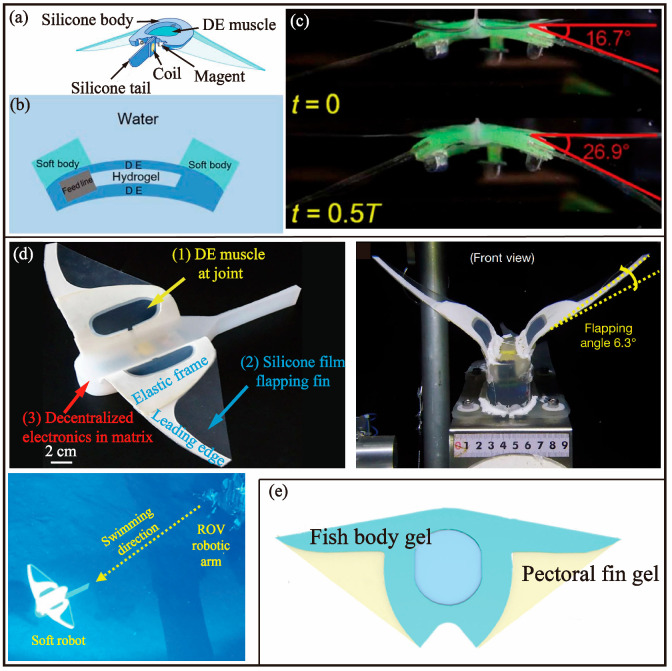
Bionic robotic fish-based MPF mode driven by DE. (**a**) Robotic fish composed of DE ionic conductive hydrogel [74]. (**b**) The soft body and the muscle laminates are deformed by the shrinking of the pre-stretched DE membranes [74]. (**c**) Bending variations of the soft body and fins [74]. (**d**) Self-powered robotic fish for deep-sea exploration [77]. (**e**) Robotic fish with hydrogel as a structural element [78].

In 2017, Li et al. [74] from Zhejiang University presented a bionic robotic fish that resembles a manta ray, as depicted in Figure 12a. The robotic fish is equipped with an airborne energy and remote-control system, which allows it to exhibit high mobility, excellent adaptability to various environments, and extended endurance. The driving mechanism is achieved by a soft electrically active structure composed of dielectric elastomer and ionic conductive hydrogel, as shown in Figure 12b. The fin is made of silicone film and the body frame is constructed from silicone. A 3.7 V battery voltage is amplified to a driving voltage of 10 kV through an onboard voltage amplification circuit. At a voltage of 9.5 kV, the maximum swimming speed was 6.4 cm/s, and a maximum bending angle of 26.9° (Figure 12c) was realized through the use of a tail fin with electromagnet. The overall weight of the bionic robotic fish is 42.5 g, and it operates within a temperature range of 0.4 °C to 74.2 °C with a maximum continuous working time of over 3 h.

The underwater environment is known for its complexity and the presence of harsh conditions such as high pressure and turbulence. These factors make it challenging for a single underwater robot to operate effectively in this environment. However, a group of underwater robots, operating together as a school, have the potential to compensate for the limitations of an individual robot [75]. This not only enables the completion of tasks that a single robot would find challenging but also enhances the efficiency of task completion. While this concept of a school of robots has promise, it also presents several challenges including formation control, group obstacle avoidance, and inter-robot communication. In 2020, Zhang et al. [76] developed a bionic robotic fish for collective control. The design comprises a flapping wing, body, steering servo motor, and steering tail. The body part of the robotic fish houses a highly compact electrical system within a centrifuge tube, while the steering tail is fabricated using laser cutting technology on an acrylic plate. The flapping wing is composed of multiple materials, including polymethyl methacrylate (PMMA), a dielectric elastomer film, a carbon grease layer, a tin foil strip, a 65-manganese steel sheet, polyethylene terephthalate (PET), and a silicon film. The maximum angle of a single robotic fish’s flapping wing is 45.22°, with a maximum speed of 6.2 cm/s. The steering tail is capable of swinging within a range of −50° to 50°, and the minimum turning radius is 0.234 m. Utilizing the global visual positioning fish school system and hybrid power control drive, the robotic fish school is able to imitate three typical cluster behaviours: a highly parallel group, a surround formation, and a torus formation.

In 2021, Li et al. [77] from Zhejiang University developed an untethered soft robotic fish for deep-sea exploration, as depicted in Figure 12d. This robotic fish measures 22 cm in length and boasts a 28 cm wingspan, and includes a battery, micro control unit, and voltage amplifier, among other electronic components, which are integrated into a silicone matrix to safeguard the airborne power, control, and drive from the high pressure present in deep-sea environments. The design of this robotic fish incorporates a DEA, which consists of two pre-stretched DE films and a flexible electrode sandwiched in the middle of these films. The DEA is located at the joint between the support frame and the flapping wing, enabling the transformation of the planar motion of the DE film into the fin flapping motion. The final test results demonstrate that the swimming speed of the robotic fish is 3.16 cm/s at a depth of 70 m, though this is slightly slower than the speed recorded at a depth of 8 m. Despite its good stability in deep-sea exploration, the robotic fish exhibits poor mobility and endurance, and its functional capabilities are relatively simple, especially in complex deep-sea conditions.

In the aquatic environment, the tissues of a majority of aquatic creatures possess a gel-like state with a water content ranging between 40–90 wt %, thus forming a soft physical structure. Compared with the traditional robot fish using rigid structures and driving materials, it can better camouflage in the surrounding environment. According to the acoustic impedance matching characteristics, a mechanical wave needs to overcome resistance when conducting in the medium. The greater the impedance, the greater the force required to push the medium. When the studied wave enters a medium from one medium during its propagation, reflection, refraction, and transmission occur at the interface of different impedance media. The larger the impedance difference between the two media, the easier this disturbance is to be detected. Despite these properties, the elastic modulus of most hydrogels is limited to the kilopascal range, which is insufficient to provide adequate structural stiffness and poses challenges in generating sustained motion. For this reason, in 2022, Zhang et al. [78] developed a novel bionic manta ray robotic fish that leverages hydrogel as its key component. The device utilized DEA encapsulated within the hydrogel, which was pre-stretched to serve as the driving unit. Upon application of voltage, the DEA generated a strain that powered the pectoral fin’s up-and-down motion, enabling the fish to achieve high-speed swimming at a velocity of up to 10 cm/s. Notably, the hydrogel utilized in the device boasted a water content of 70 wt % and an elastic modulus in the range of MPa, making it an ideal engineering material for the development of bionic robotic fish in the future.

The anatomy of a manta ray’s pectoral fins is characterized by a dichotomy between a more rigid basal region and a more pliant distal segment. As the basal portion is moved, the distal segment opposes this motion through the resistance of water [79]. According to this pectoral fin characteristic, Xu et al. [80] of Zhejiang University carried out a study to investigate the impact of the bionic shape of a robotic fish on its swimming performance. The two bionic robotic fish in Figure 12e are robotic fish without a rigid frame. The researchers used a solid–liquid interpenetrating silicone-based dielectric elastomer actuator (SIS DEA) as the driving mechanism for their bionic manta ray robotic fish, which was comprised of two layers of a solid–liquid interpenetrating silicone-based matrix that held a silicone rubber film in place. The optimized SIS DEA exhibited a strain rate of 79.8% at 20.43 kV/mm and was capable of generating a swimming speed of 5.7 mm/s in the manta ray-like robotic fish. Through both steady state and transient simulations, the researchers demonstrated that the periodic electric deformation produced by the SIS DEA generated high-speed eddy currents that pushed the manta ray-like robotic fish forward. These findings highlight the potential of the SIS DEA as a driving mechanism for bionic robotic fish with a flexible body design.

## 5. Discussion

Section 3 and Section 4, respectively, introduce prototypes based on the swimming mechanisms of BCF and MPF, but fish in nature are not binary opposites. In addition, there are also fish that use both swimming mechanisms simultaneously. For this reason, some researchers had also developed prototypes, such as the bionic box fish designed actuated by servo motors [81] and the BoxyBot actuated by DC motors [82]. This type of prototype usually relies mainly on the caudal fin as the source of thrust, and the main function of paired fins is to control direction and maintain balance. Through the mutual cooperation of the two kinds of fins, linear swimming, turning, and other motion trajectories can be achieved. Most prototypes of this type are driven by electric motors, and currently, there are few bionic robotic fish driven by smart materials that use both BCF and MPF simultaneously.

From the perspective of the driving principle, piezoelectric materials, such as PZT and macro-fibre composite, all utilize the inverse piezoelectric effect to generate driving force, which has the advantages of a large driving force and fast response speed, making them suitable as functional materials for bionic robotic fish. However, usually, piezoelectric materials generate small displacements, so additional transmission mechanisms are needed to amplify them. Therefore, there are few articles based on piezoelectric materials, and a large number of them are concentrated on prototypes that swim in BCF mode. In recent years, there are few prototypes driven by piezoelectric materials based on MPF swimming mode. However, this is also a significant research direction in the field of bionic robotic fish, so we believe that more such prototypes will be reported in the future.

For different smart materials, it is noteworthy that SMA actuators are characterized by their low cost, simple structure, corrosion resistance, high stress and strain, and ability to meet many actuator requirements for output force and output stroke. However, the recoverable strain of SMA is limited, only about 4–8%. When SMA is transformed into a spring, the strain amplitude can be effectively improved (up to 200–1000%), but the generated stress will be greatly reduced, making SMA only suitable for small robotic fish.

Table 2 shows a comparison of various types of robotic fish. Another challenge is that in order to maintain the high actuation level of SMA, it needs to produce a large temperature change within a certain period of time. Therefore, due to the limitation of temperature control, it is difficult for SMA-driven robotic fish to achieve autonomous behaviour. Although the deformation of IPMCs is large, its driving force is very low, resulting in slow swimming speed. In addition to the challenges posed by hydrodynamics, IPMCs face significant hurdles when it comes to actuation. There is a serious leakage phenomenon in the seawater plasma solution, resulting in a significant reduction in its operating range and efficiency. While piezoelectric materials such as PZT provide a large driving force, their small deformation amplitude necessitates additional structures to amplify their displacement, leading to bulky and heavy designs. High driving voltage requirements also present limitations for battery power supply technology, creating obstacles to endless flow. Furthermore, piezoelectric materials are prone to poor sealing and low efficiency, rendering them unsuitable for underwater robotics. Although dielectric elastomer (DE) actuators possess desirable characteristics such as large strain and high conversion efficiency, their driving voltage needs, which can reach several thousand volts, prevent them from achieving infinite flow or miniaturization without additional voltage amplifiers. SMA, IPMC, or hybrid actuator-driven robots are more compact and move faster than PZT robots, with SMA technology showing the most promise for practical application in ocean engineering due to factors such as use environment, output force, stroke, structure, weight, and volume. The performance parameters of different robotic fish actuators are shown in Table 3.

Table 3 shows typical BCF and MPF fish, respectively. Compared with Table 3, it can be found that although bionic robotic fish can achieve smaller sizes and lighter weights than real fish, the difference in swimming performance is too large. They still cannot reach the level of real fish in terms of swimming speed, acceleration, and other aspects. In addition, in previous studies, bionic robotic fish have varying degrees of problems, such as high driving voltage and low efficiency. At the same time, there is also a problem of low accuracy in the processing technology.

## 6. Conclusions

In this paper, we present a comprehensive overview of recent advancements in bionic robotic fish research. We succinctly summarize the current state-of-the-art on the swimming modes and hydrodynamic models of fish and delve into a detailed analysis of four prevalent actuators utilized in the development of bionic robotic fish. Our focus lies on the examination of bionic robotic fish powered by various smart materials. Subsequently, we analysed the advantages and disadvantages of different smart materials and pointed out the problems they face when applied to bionic robotic fish. We also classified and summarized various prototypes, compared them with real fish, and identified the differences between them. With the aim of furthering research and development in the field, we provide an outlook on the current challenges and future directions of bionic robotic fish research.

First of all, with the rapid development of MEMS and sensor technology, the swimming speed and direction control ability of bionic robotic fish have been greatly improved, but there is still a big gap between the swimming ability and that of real fish. For example, the maximum angular velocity of a barracuda can reach 2500 deg/s, while the current robotic fish can only reach 670 deg/s. In three-dimensional space, the bionic robotic fish often has to face frequent height changes, such as rapid diving, surfacing, and flipping, while the existing robotic fish can only complete some simple actions. In addition, the body of fish is soft in most cases, such as swimming at low speed. Only when chasing prey and avoiding tracking will it become rigid to accelerate quickly. At present, most of the bionic robotic fish are rigid structures, and there is a certain gap between them and real fish [89,93,94]. In order to make the movement ability of the bionic robotic fish approach the real fish, more practical closed-loop control methods are needed in future research to coordinate the pectoral fin, body and tail fin of the robotic fish, and to integrate and optimize the structure to complete fast and accurate manoeuvres.

Secondly, bionic machines will be widely used in extreme environments such as deep-sea exploration, and the perception of unknown environments is particularly important. At present, the research on bionic robotic fish is mostly focused on driving and control, while the research on sensing is less. It is undeniable that the perception ability of robotic fish is very limited at present, and there is a lack of visual sensors for detecting and avoiding obstacles. At present, this kind of sensor has high requirements for the underwater environment, such as the illumination brightness of the environment, the cleanliness of the water body, and the flow speed of the water. In addition, due to the impact of fish wave propulsion, head yaw is an inevitable problem for robotic fish, which will lead to large fluctuations in sensor measurement data and seriously affect the sensing accuracy, which requires proper adjustment by multi-sensor data fusion. Therefore, in the future, the bionic robotic fish will use a lot of sensing information to perceive the strange underwater environment.

In addition, the intelligence level of bionic robotic fish also needs to be improved. The self-learning ability will greatly improve the survival ability of robotic fish in the face of complex and harsh underwater environments, and the data fed back by equipped sensors will be used to adjust the behaviour. Although some researches focus on the application of reinforcement learning (RL) in robotic fish, how to develop a dynamic learning algorithm suitable for underwater environments remains to be further studied. From this perspective, the autonomy and adaptability of the bionic robotic fish system will be improved in the future.

## Figures and Tables

**Table 1:** Reference parameter and non-dimensional number.

Dimension	Reference Parameter	Non-Dimensional Number
Length	*L_ref_* = *L*	L^*^ = L/L_ref_
Velocity	*u_ref_* = *U*	u^*^ = *u*/*u_ref_*
Pressure	*p_ref_* = *ρ*$u_{ref}^{2}$	p^*^ = p/p_ref_
Time	*t_ref_* = *L_ref_/u_ref_* = *L/U*	t^*^ = t/t_ref_
Gravity	*g_ref_ = g*	g^*^ = g/g_ref_

**Table 2:** Comparison of various types of robotic fish.

Propulsion Mode	Actuator	Energy Supply	Weight (g)	Dimension (mm)	Thrust (mN)	Velocity (mm·s^−1^/BL·s^−1^)	Power Consumption (mW)
BCF	SMA	Tethered [58]	416	250 × 88 × 88	390	24.5/0.098 ^b^	
		Untethered [83]	30	146 × 34 ^a^		112/0.77	
	IPMC	Untethered [60]	100	130 × 50 × 110		30/0.23	1500
		Tethered [84]	0.76	45 × 10 × 4	0.0036	5.21/0.116	300
		Untethered [61]	180	270 × 80 × 80		120/0.45	
		Untethered [85]	16.2	96 × 24 × 25	6.5	23.6/0.246 ^b^	
	Piezoelectric	Tethered [64]	14.1	175 × 3.1 × 64		600/3.5	
		Tethered [86]	14.98	110 × 65	330	320/2.91^b^	8066
		Tethered [87]	450	360	0.71	144.45/0.4	65
		Untethered [88]		400 × 150 × 40	4.8	320/0.8 ^b^	
		Tethered [68]		305 × 286 × 286	80	280.6 ^b^/0.92	250
		Tethered [66]	1.93	60 × 28 × 20	0.243	45/0.75	0.645
	DE	Untethered [70]	115	100 × 30 × 60	25	55/0.55	1.3
MPF	SMA	Untethered [71]		126 × 234 × 10	15	45/0.36	
		Untethered [89]		225 × 330 × 50		58/0.25	
	IPMC	Untethered [59]	55.3	80 × 180 × 25	5	4.2/0.053	483
		Untethered [72]	55	110 × 210 × 25		7.4/0.067	2500
		Tethered [73]	67.4	177 × 57	0.4	28/0.16	4000
	DE	Tethered [74]	42.5	185 × 220 × 40	18	135/1.45	2.43
		Untethered [77]	450	220 × 280		51.9/0.45	
		Tethered [90]	4.4	150 × 35 × 0.75		37.2/0.25	920

^a^ The value is estimated by the scale in the paper. ^b^ The value is calculated by the formula *v*_BL_ = *v*/*L*. *L* is the body length of bionic robotic fish.

**Table 3:** Tuna and manta.

Species	Propulsion Mode	Weight (kg)	Size (m)	Max Velocity (m/s)	Reference
Tune	BCF	600–700	3.5	44	[91]
Manta	MPF	300	0.6–7	0.83	[92]

## Data Availability

Not applicable.

## List of Abbreviations

**Abbreviation**	**Definition**
AUVs	Autonomous Underwater Vehicles
BCF	Body and/or Caudal Fin Swing
MPF	Median and/or Paired Fin Swing
EBT	Elongated Body Theory
WPT	Wave Plate Theory
SMA	Shape Memory Alloys
PZT	Lead Zirconate Titanate
IPMC	Ionic Polymer Metal Composites
CPG	Central Pattern Generator
CFD	Computational Fluid Dynamics
N–S	Navier–Stokes
LAEBT	Large Amplitude Elongated Body Theory
SMA	Shape Memory Alloy
SME	Shape Memory Effect
PVC	Polyvinyl Chloride
ABS	Acrylonitrile-Butadiene-Styrene
CFRP	Carbon Fibre-Reinforced Polymer
MFC	Macro-Fibre Composite
DEA	Dielectric Elastomer Actuator
PMMA	Polymethyl Methacrylate
PDMS	Polydimethylsiloxane
PET	Polyethylene Terephthalate
SIS DEA	Silicone-Based Dielectric Elastomer Actuator
DE	Dielectric Elastomer
RL	Reinforcement Learning
